# Diagnostic performance of Node Reporting and Data System (Node-RADS) for assessing mesorectal lymph node in rectal cancer by CT

**DOI:** 10.1186/s12885-024-12487-0

**Published:** 2024-06-11

**Authors:** Yue Niu, Lu Wen, Yanhui Yang, Yi Zhang, Yi Fu, Qiang Lu, Yu Wang, Xiao Yu, Xiaoping Yu

**Affiliations:** 1 Department of Diagnostic Radiology, the Affiliated Cancer Hospital of Xiangya School of Medicine, Central South University/Hunan Cancer Hospital, Changsha, Hunan 410013 China; 2 Department of Diagnostic Radiology, Graduate Collaborative Training Base of Hunan Cancer Hospital, Hengyang Medical School, University of South China, Hengyang, Hunan 421001 China; 3 Medical department, the Affiliated Cancer Hospital of Xiangya School of Medicine, Central South University/Hunan Cancer Hospital, Changsha, Hunan 410013 China; 4 Clinical and Technical Support, Philips Healthcare, Shanghai, 200072 China

**Keywords:** Rectal cancer, Lymph node, Computed tomography, X-ray, Node-RADS

## Abstract

**Background:**

To compare the diagnostic performance of the Node-RADS scoring system and lymph node (LN) size in preoperative LN assessment for rectal cancer (RC), and to investigate whether the selection of size as the primary criterion whereas morphology as the secondary criterion for LNs can be considered the preferred method for clinical assessment.

**Methods:**

Preoperative CT data of 146 RC patients treated with radical resection surgery were retrospectively analyzed. The Node-RADS score and short-axis diameter of size-prioritized LNs and the morphology-prioritized LNs were obtained. The correlations of Node-RADS score to the pN stage, LNM number and lymph node ratio (LNR) were investigated. The performances on assessing pathological lymph node metastasis were compared between Node-RADS score and short-axis diameter. A nomogram combined the Node-RADS score and clinical features was also evaluated.

**Results:**

Node-RADS score showed significant correlation with pN stage, LNM number and LNR (Node-RADS of size-prioritized LN: r = 0.600, 0.592, and 0.606; Node-RADS of morphology-prioritized LN: r = 0.547, 0.538, and 0.527; Node-RADSmax: r = 0.612, 0.604, and 0.610; all *p* < 0.001). For size-prioritized LN, Node-RADS achieved an AUC of 0.826, significantly superior to short-axis diameter (0.826 vs. 0.743, *p* = 0.009). For morphology-prioritized LN, Node-RADS exhibited an AUC of 0.758, slightly better than short-axis diameter (0.758 vs. 0.718, *p* = 0.098). The Node-RADS score of size-prioritized LN was significantly better than that of morphology-prioritized LN (0.826 vs. 0.758, *p* = 0.038). The nomogram achieved the best diagnostic performance (AUC = 0.861) than all the other assessment methods (*p* < 0.05).

**Conclusions:**

The Node-RADS scoring system outperforms the short-axis diameter in predicting lymph node metastasis in RC. Size-prioritized LN demonstrates superior predictive efficacy compared to morphology-prioritized LN. The nomogram combined the Node-RADS score of size-prioritized LN with clinical features exhibits the best diagnostic performance. Moreover, a clear relationship was demonstrated between the Node-RADS score and the quantity-dependent pathological characteristics of LNM.

**Supplementary Information:**

The online version contains supplementary material available at 10.1186/s12885-024-12487-0.

## Introduction

Colorectal cancer is the most prevalent gastrointestinal tumor, with China accounting for 28.2% of the total number of cases and 28.1% of the total number of deaths worldwide, ranking first in the world [1]. Rectal cancer (RC) comprises over one-third of all colorectal cancer cases [2]. The occurrence of lymph node metastasis (LNM) in RC patients is highly correlated with poor clinical prognosis and tumor recurrence [3]. Patients with LNM can benefit from total neoadjuvant therapy (TNT), considerably reducing distant metastasis and improving disease-free survival rates [4]. However, over-treatment of the lymph node (LN) stage may lead to genitourinary system damage and other consequences [5, 6]. Therefore, the preoperative identification of LN status in RC patients is crucial for tailoring treatment strategies.

Contrast-enhanced computed tomography (CE-CT) is recommended by the National Comprehensive Cancer Network (NCCN) guidelines as appropriate preoperative imaging workups for RC [7]. Currently, the assessment of LN size and morphology remains the primary method for radiologists to determine LNM. While node size is the major evaluation criterion, benign and malignant LNs overlap in size [8]. In CT images, LNs with a diameter greater than 10 mm are usually judged to be malignant. However, it has been reported that the prevalence of pathological LNM in mesorectal LNs smaller than 5 mm is at least 15% [9]. Nowadays, some studies have utilized various image criteria based on morphology [10, 11]. Li et al. suggested that the presence of LN necrosis, irregular margins and heterogeneity enhancement could be effectively used to assess the LN status of stage T1 colorectal cancer [12]. Therefore, both size and morphological features are important imaging indicators for assessing LN status. Previously, due to a lack of consensus on appropriate criteria to evaluate LN involvement, the accuracy of CT in detecting LNM has been consistently suboptimal, with an overall accuracy ranging from 59 to 68% [13]. In this circumstance, accurate and standardized preoperative assessment of the LN status in RC is of paramount importance to provide optimal treatment.

With the increasing adoption of the Reporting and Data Systems (RADS) in various clinical scenarios, there has been attempts at standardizing the reporting of oncological scans [14, 15]. Recently, Elsholtz et al. introduced a three-level flowchart-based LN comprehensive scoring system (Node-RADS), with levels 1 and 2 addressing size and configuration criteria respectively and level 3 providing the results of Node-RADS score. The Node-RADS scoring system involves a radiological assessment of LNM on standardized CT scans, making it applicable to various tumor types, anatomical sites, as well as regional and non-regional LNs [16]. Since its introduction in 2021, Node-RADS has been validated only in prostate cancer, bladder cancer, lung cancer, colon cancer and cervical cancer, showing promising results [17–21]. However, there has been no prior investigation into the role of Node-RADS in RC.

Therefore, this study aims to compare the diagnostic performance of the Node-RADS scoring system with the size of LN on preoperative assessment of LNM status in RC. We also explore whether the selection of size as the primary criterion whereas morphology as the secondary criterion for LNs can be considered the preferred method for clinical evaluation of LNM status in RC or not.

## Materials and methods

### Patients

**Fig. 1:**
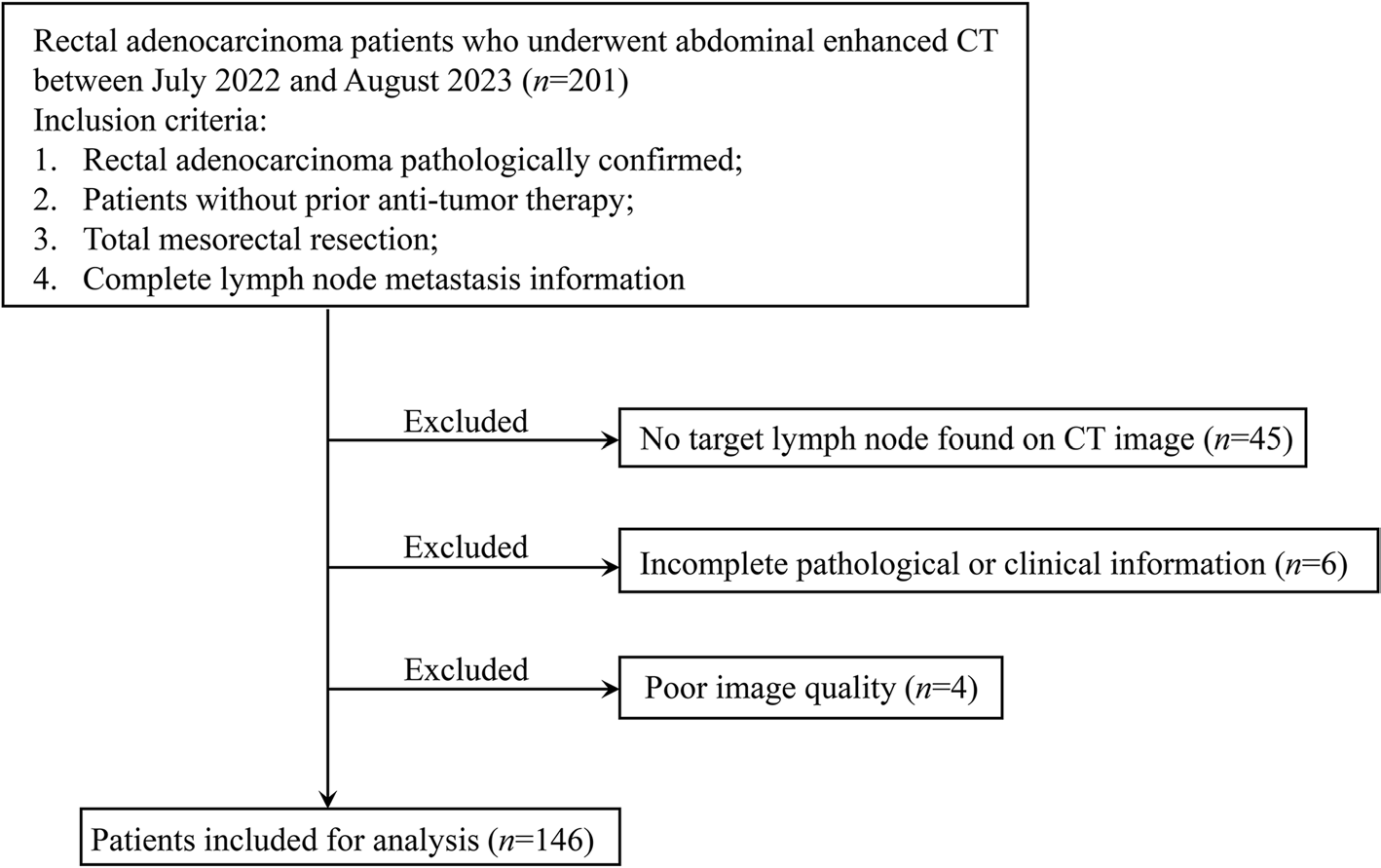
A flow diagram of patient recruitment, including inclusion and exclusion criteria

The study was approved by the Ethics Committee of Hunan Cancer Hospital and a waiver of informed consent was obtained. We retrospectively evaluated 201 patients at our hospital between July 2022 and August 2023.Eligible patients were those with pathologically confirmed rectal adenocarcinoma who underwent radical resection with LN dissection and who had received abdominal enhanced CT imaging before surgery. A flow diagram of the recruitment pathway, including inclusion and exclusion criteria, is shown in Fig. 1.

A total of 146 patients were ultimately included in our study. All patients underwent total mesorectal resection. The clinical data were collected from medical records, including age, sex, tumor location, pretreatment carcinoembryonic antigen (CEA), pretreatment carbohydrate antigen 19–9 (CA19-9), tumor histologic grade, maximum tumor diameter, clinical T (cT) stage and pathological LNM status. Tumor histologic grade was based on the poorest differentiated components observed during endoscopic examination [12]. Two pathologists specializing in colorectal cancer carefully examined the rectal specimens to harvest LNs. The pathological reports were based on the AJCC 8th TNM staging system [22]. We referred to the histopathological assessment of LNM as the gold standard. The number of harvested LNs, the number of determined positive LNs (LNM number), and the lymph node ratio (LNR) were recorded for each patient. LNR indicates the ratio of LNM number to the number of harvested LNs. The pathologically negative group (pN-) was defined as patients with no regional LNM. The pathologically positive group (pN +) was defined when patients’ number of regional LNM was greater than or equal to one.

### Image acquisition and analysis

CT imaging data were acquired using a dual-layer detector spectral CT scanner (IQon spectral CT, Philips Healthcare, The Netherlands). The patients were scanned craniocaudally in the supine position, and the scan range comprised the upper edge, including the diaphragm, and the lower, surpassing the symphysis pubis. The scanning parameters were as follows: tube voltage, 120 kVp; automated current modulation; pitch, 0.609; collimation, 64 × 0.625 mm; rotation time, 0.5 s. The non-enhanced scans were performed first. Then the iodinated nonionic contrast media (ioversol, 320 mg I/ml) was administered via peripheral vein at a dosage of 1.5 ml/kg with the flow rate of 2.5 ml/s, then 30 ml saline injected at the same rate. The bolus-tracking technique was used to control individual contrast injection timing. The portal venous-phase scanning automatically began 25 s after the trigger attenuation threshold (100 HU) was reached at the level of the descending aorta. After scanning, the conventional CT images and spectral base images (SBI) were simultaneously generated, with slice thickness of 1 mm and slice increment of 1 mm.

### Selection of lymph nodes

**Fig. 2:**
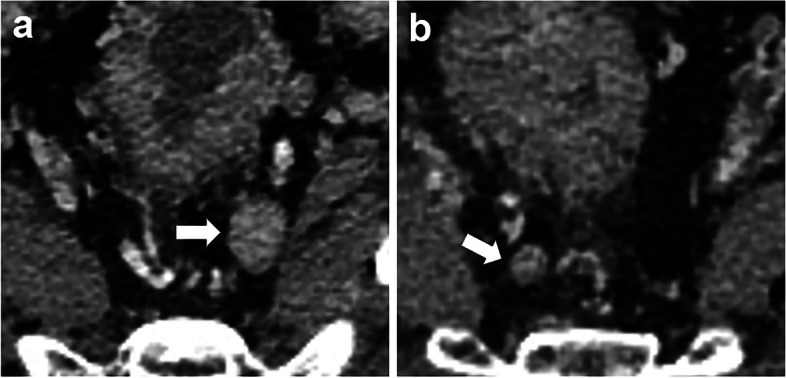
portal venous phase CT images of a 52-year-old male with moderately differentiated pathologic T3N- rectal adenocarcinoma. **a** The size-prioritized lymph node (indicated by a white arrow) exhibits a short-axis diameter of 12 mm, featuring smooth margins, homogeneous enhancement, and an elliptical shape. It has been assigned a Node-RADS score of 2. **b** The morphology-prioritized lymph node (indicated by a white arrow) demonstrates a short-axis diameter of 6 mm, displaying clear borders, heterogeneous enhancement, and an elliptical shape. It has been assigned a Node-RADS score of 3. The Node-RADSmax score for this patient is 3

**Fig. 3:**
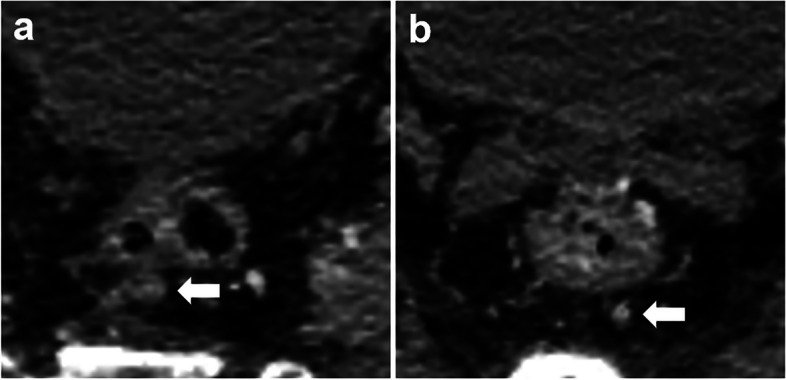
portal venous phase CT images of a 68-year-old male with moderately differentiated pathologic T3N + rectal adenocarcinoma. **a** The size-prioritized lymph node (indicated by a white arrow) exhibits a short-axis diameter of 5 mm, with ill-defined borders, heterogeneous enhancement, and an elliptical shape. It has been assigned a Node-RADS score of 4. **b** The morphology-prioritized lymph node (indicated by a white arrow) shows a short-axis diameter of 4 mm, with unclear borders, heterogeneous enhancement, and an irregular shape. It has been assigned a Node-RADS score of 3. The Node-RADSmax score for this patient is 4

On the portal venous-phase image, LNs inside the mesorectum and around the superior rectal artery were examined. The size-prioritized LN was defined as the LN with the longest short-axis diameter within the aforementioned regions. Radiologists firstly identified the size-prioritized LN in CT images of each RC patient, then selected the LN with the highest Node-RADS score from the remaining nodes with any of the morphological characteristics, defining it as morphology-prioritized LN, and recorded their short-axis diameter and Node-RADS score **(**Figs. 2 and 3**)**. The morphological characteristics are as follows: 1). Heterogeneous texture, including necrosis, calcification or mucinous; 2). Irregular LN margins; 3). LN is round rather than elliptical in shape [16, 23]. All LNs were analyzed by two trained radiologists (reader 1 with 2 years, reader 2 with more than 10 years of experience in abdominal imaging) who were blind to the postoperative pathological results. A third radiologist (reader 3, with more than 20 years of experience in abdominal imaging) was invited if there were any disagreements. All images were evaluated with commercially available DICOM software (RadiAnt DICOM Viewer).

### Node-RADS score assessment

The score assessment of LNs was performed according to Node-RADS recommendations, guided by a three-level flowchart (Supplementary Figure S1). Specifically, LNs were limited to the mesorectal region in this study, thus categorized as "enlarged" when the short-axis diameter was ≥ 5 mm. Node-RADS score was respectively applied to the size-prioritized LN and the morphology-prioritized LN for each RC patient. Node-RADSmax was defined as the higher Node-RADS score between the size-prioritized LN and the morphology-prioritized LN in each patient.

### Statistical analyses

The patients were divided into the pN- and pN + groups based on pathological LNM result. The agreement between the two radiologists was evaluated with Cohen’s kappa statistics. Shapiro–Wilk test was performed to determine the normality of the data distribution. Continuous variables were expressed as means with standard deviations (SD) or medians with interquartile ranges (IQR) where appropriate. Categorical variables were presented as counts and percentages. The t test or Mann–Whitney U test was used for continuous variables or chi-square for categorical variables. Spearman correlation analysis was performed to survey the correlation of Node-RADS score to the quantity-dependent pathological characteristics of LNM. The receiver operating characteristic (ROC) curves with area under the curve (AUC) were performed to assess the predictive ability of pN status. The Delong method was used to compare the AUCs of different predictors. Sensitivity, specificity, accuracy, positive predictive value (PPV), and negative predictive value (NPV) for pN prediction were calculated according to the optimal threshold using the Youden index. Subsequently, variables that achieved significance at *p* < 0.05 in univariate analysis of the correlation with pN status were entered into the multivariate analysis to determine the predictors included in the Clinical-Node-RADS nomogram, using a stepwise logistic regression model with Akaike information criterion (AIC) [24]. The goodness-of-fit of the nomogram was evaluated by Hosmer–Lemeshow test, whereas its accuracy was evaluated by calibration curve. All the statistical analyses were performed with SPSS STATISTICS (IBM, version 25.0; Armonk, NY, USA) and R software (version 3.6.1). All tests were two sided, and a *p* value of 0.05 or less was considered statistically significance.

## Results

### Clinical characteristics of study population

Table1 summarizes the characteristics of the 146 eligible patients enrolled in our study. There were significant differences in terms of tumor location, CA19-9, cT stage and histologic grade between the pN + and pN- groups.

**Table 1:** Clinical characteristics of the pN + and pN- groups

Characteristic	Total (*n* = 146)	pN- (*n* = 64)	pN + (*n* = 82)	*p*
Age (years)	60.2 ± 10.6	59.9 ± 10.4	60.4 ± 10.8	0.772
Sex (No.)				0.851
Male	90(61.6%)	40(62.5%)	50(61.0%)	
Female	56(38.4%)	24(37.5%)	32(39.0%)	
Location (No.)				0.043
Upper	36(24.7%)	10(15.6%)	26(31.7%)	
Middle	65(44.5%)	29(45.3%)	36(43.9%)	
Lower	45(30.8%)	25(39.1%)	20(24.4%)	
CEA (mg/L)	3.44 [1.56,8.93]	3.79 [1.48,8.82]	3.20 [1.77,9.12]	0.730
CA19-9 (U/mL)	8.52 [5.47,15.5]	7.41 [4.11,13.2]	9.08 [6.19,19.2]	0.030
cT stage (No.)				< 0.001
T1	4(2.7%)	3(4.7%)	1(1.2%)	
T2	42(28.8%)	30(46.9%)	12(14.6%)	
T3	100(68.5%)	31(48.4%)	69(84.1%)	
Histologic grade (No.)				< 0.001
Well differentiated	1(0.7%)	1(1.6%)	0(0.0%)	
Moderately differentiated	120(82.2%)	61(95.3%)	59(72.0%)	
Poorly differentiated	25(17.1%)	2(3.1%)	23(28.0%)	
Maximum tumor diameter (cm)	4.50 [3.50,5.80]	4.50 [3.80,5.80]	4.50 [3.50,5.75]	0.455

Continuous data are shown as mean ± standard deviation (SD) or median (interquartile range, IQR), and categorical data as number (percentage)

*Abbreviations: pN-* patients with no lymph node metastases confirmed by pathology, *pN* + patients with one or more lymph node metastases confirmed by pathology, *CEA* Carcinoembryonic antigen, CA19-9 Cancer antigen 19–9, *cT stage* clinical T stage

### Differences in imaging characteristics between the pN + and pN- groups

Inter-observer agreement (Cohen’s kappa) of Node-RADS1 (Node-RADS score of the size-prioritized LN), Node-RADS2 (Node-RADS score of the morphology-prioritized LN), Node-RADSmax were 0.78, 0.71 and 0.82, respectively. Between the pN + and pN- groups, there were significant differences in the short-axis diameter and Node-RADS score of both the size-prioritized LN and morphology-prioritized LN (Table 2).

**Table 2:** Short-axis diameter and Node-RADS Score of lymph nodes in the pN + and pN- groups

Predictor	Total (*n* = 146)	pN- (*n* = 64)	pN + (*n* = 82)	*p* value
Short-axis diameter of LN1 (mm)	6.6[5.3,8.4]	5.5[4.8,6.9]	7.7[5.9,10.3]	< 0.001
Short-axis diameter of LN2 (mm)	4.7[3.9,6.1]	4.3[3.7,5.1]	5.4[4.3,6.6]	< 0.001
Node-RADS1				< 0.001
1	7 (4.8%)	7(11.0%)	0(0.0%)	
2	25 (17.1%)	19(29.7%)	6(7.3%)	
3	42 (28.8%)	26(40.6%)	16(19.5%)	
4	38 (26.0%)	10(15.6%)	28(34.2%)	
5	34 (23.3%)	2(3.1%)	32(39.0%)	
Node-RADS2				
1	1 (0.7%)	1(1.6%)	0(0.0%)	< 0.001
2	56 (38.3%)	38(59.4%)	18(21.9%)	
3	42 (28.8%)	18(28.1%)	24(29.3%)	
4	27 (18.5%)	6(9.3%)	21(25.6%)	
5	20 (13.7%)	1(1.6%)	19(23.2%)	
Node-RADSmax				< 0.001
1	1(0.6%)	1(1.6%)	0(0.0%)	
2	26(17.8%)	21(32.8%)	5(6.1%)	
3	43(29.5%)	28(43.7%)	15(18.3%)	
4	33(22.6%)	11(17.2%)	22(26.8%)	
5	43(29.5%)	3(4.7%)	40(48.8%)	

*Abbreviations*: *pN-* patients with no lymph node metastases confirmed by pathology, *pN* + patients with one or more lymph node metastases confirmed by pathology; LN1: the size-prioritized lymph node; LN2: the morphology-prioritized lymph node; Node-RADS1: Node-RADS score of the size-prioritized lymph node; Node-RADS2: Node-RADS score of the morphology-prioritized lymph node; Node-RADSmax: taking the higher score between Node-RADS1 and Node-RADS2 as the Node-RADSmax score

### Correlation of Node-RADS score to pN stage, LNM number and LNR

The pN results included N0 (*n* = 67), N1a (*n* = 19), N1b (*n* = 30), N2a (*n* = 19), and N2b (*n* = 11) stages. Additionally, N1c stage was found in 13 patients, which was accompanied by other pN stages (N1a, N1b, N2a, or N2b). Considering that N1c represents tumor deposits and is unrelated to the number of positive LNs, N1c wasn't included the correlation analysis. The LNM number was 3.55 ± 3.34 (range:1 ~ 22), and LNR was 0.31 ± 0.23 (range: 0.06 ~ 0.96) in the pN + group.

Node-RADS score showed significant correlation with pN stage (i.e., N1a, N1b, N2a, and N2b), LNM number and LNR (Node-RADS1: r = 0.600, 0.592, and 0.606; Node-RADS2: r = 0.547, 0.538, and 0.527; Node-RADSmax: r = 0.612, 0.604, and 0.610; respectively, all *p* < 0.001).

### Diagnostic performance of Node-RADS score according to different cut-offs

By setting a higher cut-off (from 1 to 4) for Node-RADS1 score, the specificity and PPV increased from 10.9% to 96.9% and from 59.0% to 94.1%, respectively. Conversely, the sensitivity and NPV decreased from 100% to 39.0% and from 100% to 55.4%, respectively (Supplementary Table S1). Similar trends were recorded in cut-offs for Node-RADS2 (Supplementary Table S2) and Node-RADSmax (Supplementary Table S3). Interestingly, score > 3 could be considered as the best cut-off for both Node-RADS1 and Node-RADSmax, based on the highest accuracy and the optimal Youden index (76.7%, 73.2%, 81.3% vs. 76.7%, 75.6%, 78.1%), while the best cut-off score for Node-RADS2 was > 2, with corresponding accuracy, sensitivity and specificity of 70.5%, 78.0% and 60.9%, respectively.

### Comparison of short-*axis* diameter and Node-RADS Score for predicting pN Status

**Fig. 4:**
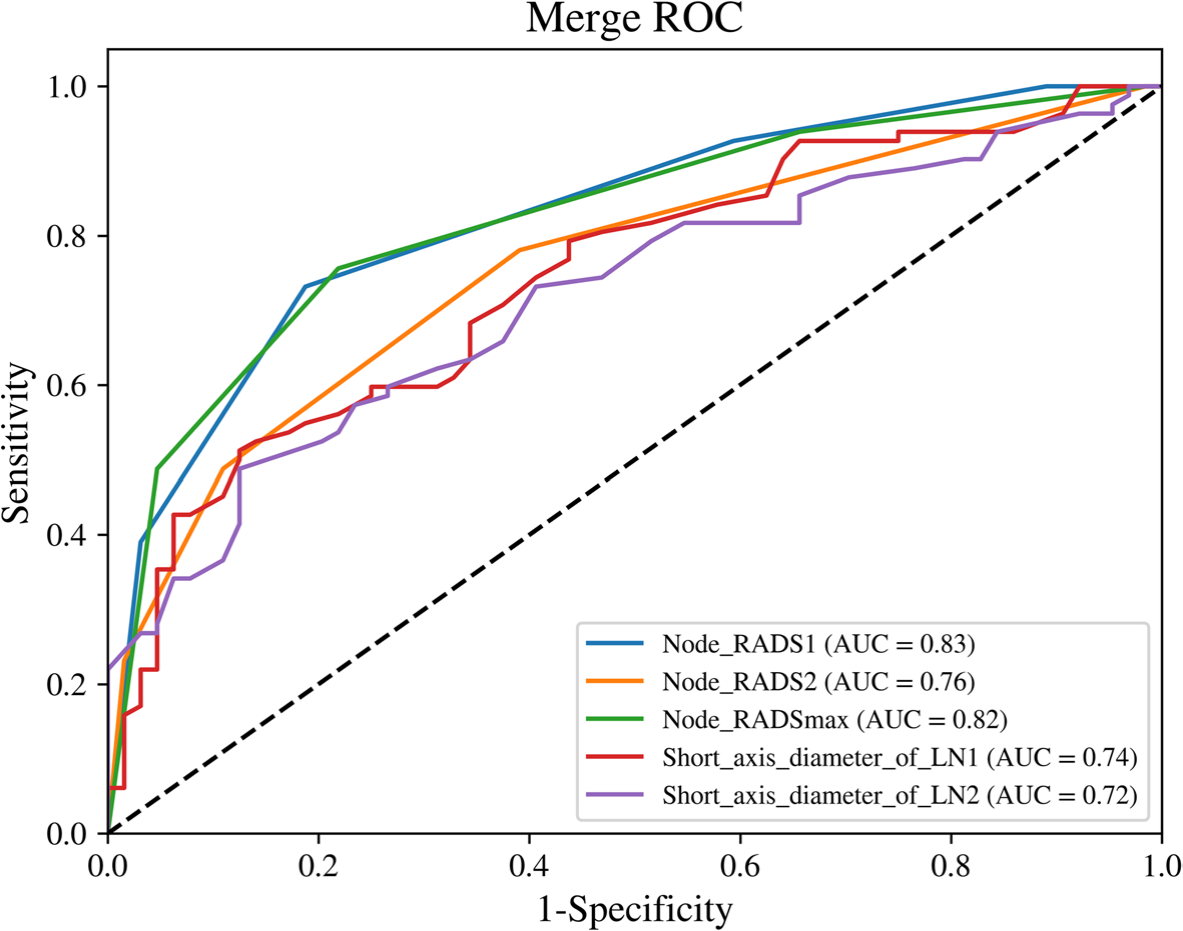
Receiver operating characteristic curves (ROC) for predicting pN status based on the short-axis diameter and Node-RADS Score of the size-prioritized LN and the morphology-prioritized LN. Abbreviations: LN1: the size-prioritized lymph node; LN2: the morphology-prioritized lymph node; Node-RADS1: Node-RADS score of the size-prioritized lymph node; Node-RADS2: Node-RADS score of the morphology-prioritized lymph node; Node-RADSmax: taking the higher score between Node-RADS1 and Node-RADS2 as the Node-RADSmax score

Table 3 and Fig. 4 shows the diagnostic performance of short-axis diameter and Node-RADS Score for predicting pN status. Generally, Node-RADS had higher AUC values than short-axis diameter, no matter based on size-prioritized LN or morphology-prioritized LN. For size-prioritized LN, Node-RADS1 was significantly superior to short-axis diameter (0.826 vs. 0.743, *p* = 0.009). For morphology-prioritized LN, Node-RADS2 exhibited an AUC of 0.758, which tended to be significantly better than short-axis diameter (0.758 vs. 0.718, *p* = 0.098). In addition, compared to morphology-prioritized LN, size-prioritized LN has a larger AUC value, whether it was based on short-axis diameter or Node-RADS Score. Specifically, Node-RADS1 was significantly better than Node-RADS2 (0.826 vs. 0.758, *p* = 0.038) and similar to Node-RADSmax (0.826 vs. 0.823, *p* = 0.780). In regard to short-axis diameter, size-prioritized LN was slightly higher than morphology-prioritized LN (0.743 vs. 0.718, *p* = 0.398).

**Table 3:** Performance and comparison of short-axis diameter and Node-RADS Score for predicting pN status

Predictor	Cutoff	AUC (95%CI)	*p* value	Sensitivity	Specificity	PPV	NPV	Accuracy
Short axis diameter of LN1	> 7.6 mm	0.743(0.664–0.823)	0.009*	51.20%	87.50%	84.00%	58.30%	67.10%
Short axis diameter of LN2	> 5.5 mm	0.718(0.636–0.800)	0.005*	48.80%	87.50%	83.30%	57.10%	65.80%
Node-RADS1	> 3	0.826(0.759–0.893)	Reference	73.20%	81.30%	83.30%	70.30%	76.70%
Node-RADS2	> 2	0.758(0.681–0.835)	0.038*	78.00%	60.90%	71.90%	68.40%	70.50%
Node-RADSmax	> 3	0.823(0.755–0.891)	0.780	75.60%	78.10%	81.60%	71.40%	76.70%

Abbreviations: AUC: area under the receiver operating characteristic curve; CI: Confidential Interval; PPV: positive predictive value; NPV: negative predictive value; LN1: the size-prioritized lymph node; LN2: the morphology-prioritized lymph node; Node-RADS1: Node-RADS score of the size-prioritized lymph node; Node-RADS2: Node-RADS score of the morphology-prioritized lymph node; Node-RADSmax: taking the higher score between Node-RADS1 and Node-RADS2 as the Node-RADSmax score

*p* values were obtained by comparing the AUCs with that of Node-RADS1

^*^ *p* < 0.05

### Development of Clinical-Node-RADS nomogram for predicting pN Status

**Fig. 5:**
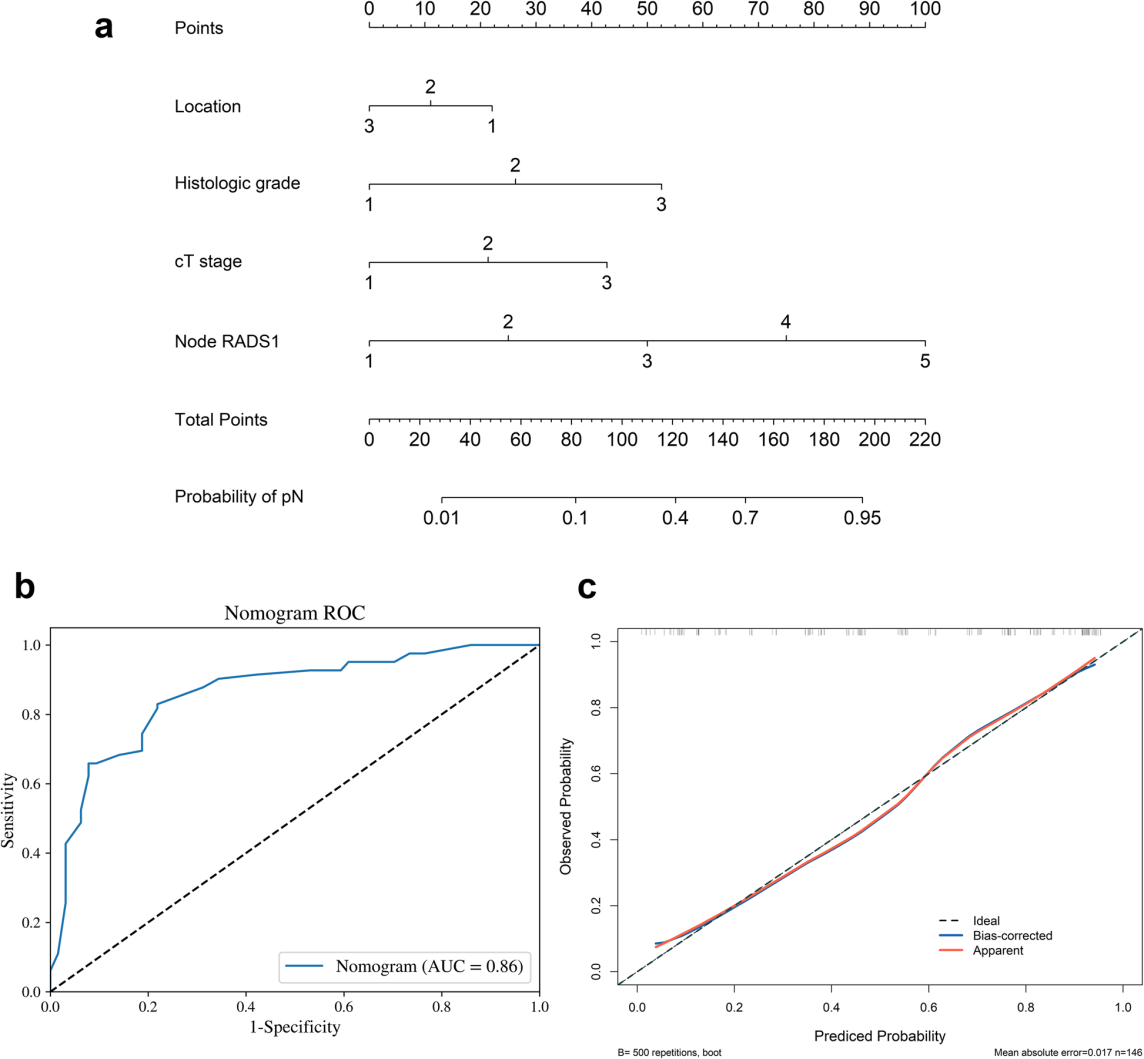
Construction and evaluation of the Clinical-Node-RADS nomogram. **a** The Clinical-Node-RADS nomogram for predicting pN status. **b** Receiver operating characteristic curves (ROC) of Clinical-Node-RADS nomogram. **c** The orange line represents the initial performance of the nomogram without any corrections. The blue line illustrates the nomogram’s calibration after addressing the observed bias. The diagonal black dotted line signifies a scenario in which the predicted probabilities perfectly align with the observed probabilities

**Fig. 6:**
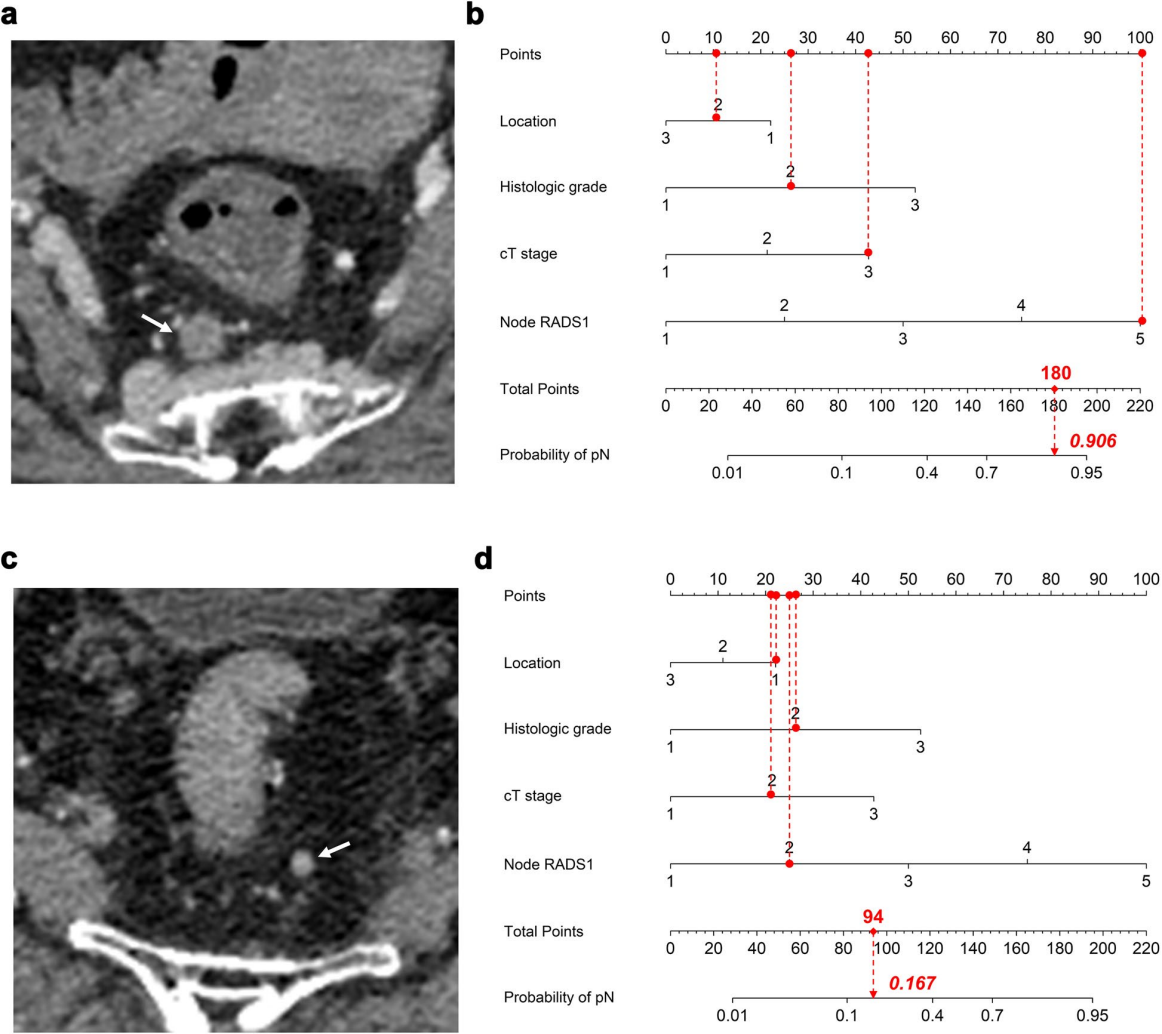
Examples of application of the Clinical-Node-RADS nomogram to predict probability of pN + in rectal cancer. Each nomogram shows value of each predictor on axis for variable and its corresponding score on points scale (as designated by red solid circles connected by red dashed lines). When points for all variables were added, total scores and corresponding risk probability were superimposed on scales showing total points and probability of pN + . **a-b** a 71-year-old female with moderately differentiated cT3 middle rectal adenocarcinoma. **a** On the portal venous phase CT image, the size-prioritized lymph node (indicated by a white arrow) exhibits a short-axis diameter of 9 mm, with ill-defined borders, focal necrosis, and a spherical shape without fatty hilum. It has been assigned a Node-RADS score of 5. When points for individual predictors were added, total points were 180. **b** Clinical-Node-RADS nomogram revealed that probability of patient with pN + was 0.906. Histopathological examination confirmed the patient's lymph node status was pN + . **c-d** a 76-year-old male with moderately differentiated cT2 upper rectal adenocarcinoma. **c** On the portal venous phase CT image, the size-prioritized lymph node (indicated by a white arrow) exhibits a short-axis diameter of 4 mm, featuring smooth margins, homogeneous enhancement and a spherical shape without fatty hilum. It has been assigned a Node-RADS score of 2. When points for individual predictors were added, total points were 94. **d** Clinical-Node-RADS nomogram revealed that probability of patient with pN + was 0.167. Histopathological examination confirmed the patient's lymph node status was pN-

To facilitate clinical use, a total of 13 clinical and imaging predictors were evaluated by univariate and multivariate logistic regression analyses (Table 4), to development a Clinical-Node-RADS nomogram for predicting pN Status. Eventually, the Node-RADS1 score, cT stage, histologic grade and tumor location were retained in the Clinical-Node-RADS nomogram (Fig. 5a). The nomogram predicts the probability of pN + , within a range of 0 to 1. A probability close to 1 indicated high odds of pN + .In regard to predict pN Status, the AUC value of the Clinical-Node-RADS nomogram was 0.861 when the cut-off of probability was 0.581 based on maximal Youden index, which significantly outperformed Node-RADS1 (0.861 vs. 0.826, *p* < 0.001), as shown in Fig. 5b. The calibration curve of the Clinical-Node-RADS nomogram is shown in Fig. 5c. Hosmer–Lemeshow test identified good calibration in the Clinical-Node-RADS nomogram (*p* > 0.05). RC patients could benefit from this prediction model. When the probability exceeded 0.581, we classified the patient's LN status as pN + . If it was below 0.581, we considered the status as pN-. Two examples of application of the Clinical-Node-RADS nomogram to predict the probability of pN + are shown in Fig. 6.

**Table 4:** Univariate and stepwise multivariate logistic regression analysis of predictors in predicting pN status

Predictor	Univariate logistic regression				Multivariate logistic regression			
	OR	95% CI lower	95% CI upper	*p* value	OR (95%CI)	95% CI lower	95% CI upper	*p* value
Age	1.00	0.97	1.04	0.771				
Sex	1.07	0.54	2.09	0.851				
CEA	1.01	0.98	1.03	0.559				
CA19-9	1.02	1.00	1.05	0.055				
Location	0.56	0.36	0.89	0.014	0.61	0.34	1.08	0.089
Histologic grade	12.43	2.83	54.61	< 0.001	3.29	0.67	16.25	0.144
Maximum tumor diameter	0.97	0.79	1.18	0.754				
cT stage	4.67	2.27	9.63	< 0.001	2.63	1.12	6.22	0.027
Short axis diameter of LN1	1.47	1.23	1.76	< 0.001				
Short axis diameter of LN2	1.92	1.43	2.59	< 0.001				
Node-RADS1	3.87	2.47	6.04	< 0.001	3.10	1.92	5.01	< 0.001
Node-RADS2	3.01	1.97	4.60	< 0.001				
Node-RADSmax	3.84	2.48	5.94	< 0.001				

*Abbreviations*: *OR* Odds Ratio, *CI* Confidential Interval, *pN* pathological result of lymph node metastasis, *CEA* Carcinoembryonic antigen, *CA19-9* Cancer antigen 19–9, *cT stage* clinical T stage, LN1: the size-prioritized lymph node; LN2: the morphology-prioritized lymph node; Node-RADS1: Node-RADS score of the size-prioritized lymph node; Node-RADS2: Node-RADS score of the morphology-prioritized lymph node; Node-RADSmax: taking the higher score between Node-RADS1 and Node-RADS2 as the Node-RADSmax score

## Discussion

Both morphology and size of LN are common imaging indicators to evaluate LNM. Based on this consideration, the Node-RADS scoring system was recently introduced to assessing the likelihood of LNM. Node-RADS system encompasses about the size and morphology information of LN. While Node-RADS and size have proven to be helpful in determining LNM, there has been no report on comparing the diagnostic performance of these imaging approaches in RC. In this study, we compared the diagnostic performance of Node-RADS and size of LN in detecting regional LNM in RC, and found that Node-RADS is superior to size. In this study, we also explored which type of LN should be prioritized when applying the above-mentioned imaging approaches, and found the size-prioritized LN is a better choice than the morphology-prioritized LN. Additionally, we also demonstrated obvious relationship of Node-RADS score with the quantity-dependent pathological characteristics of LNM.

Generally, Node-RADS scoring system outperformed short-axis diameter on the diagnosis of LNM in our study, regardless of whether size-prioritized or morphology-prioritized LNs were assessed. Our findings may be due to the Node-RADS scoring system integrates information on both size and morphological abnormalities. According to the scoring principles of Node-RADS system, the contribution of LN size and morphological abnormalities on the score is cumulative. In other words, the larger the size and the greater the number of morphological abnormalities, the higher the score, indicating a higher likelihood of malignancy. This is consistent with common experience in clinical practice and previous reports [25], as larger LN size combining with a greater number of morphological abnormalities indicate a higher possibility of LNM.

According to the Node-RADS scoring system, if multiple abnormal LNs exist in the same LN group or region, the LN with the highest score should be reported, unless the TNM stage or therapy depends on the number of LNs with metastasis [16]. Evaluating each individual lymph node one by one is undoubtedly time-consuming, labor-intensive and inefficient. Theoretically, the largest LN may not necessarily have the highest number of morphological abnormalities (namely, the highest Node-RADS score), and vice versa. Thus, the choice between size-prioritized LN and morphology-prioritized LN as the preferred one for assessing pN status is a topic worthy of exploration. Generally, the size-prioritized LN exhibited better performance than morphology-prioritized LN in the present study, especially when applicating the Node-RADS scoring system and short-axis diameter. Specifically, the Node-RADS score from size-prioritized LN was the independent predictor of pN status in multivariate regression analysis and it exhibited the best diagnostic performance (AUC = 0.826) among all the individual predictors. The above findings highlight that the application of Node-RADS to the prediction of LNM in RC should focus on the LN with the largest size. Similarly, Maggialetti et al. reported that the Node-RADS score based on the largest LN achieved a significant correlation with the pathological status of LNM in colon cancer [20]. These observations reinforce the notion that larger LN can be more accurately characterized through the analysis of their size and morphological features [26].

In recent years, the emergence of radiomics has provided an objective quantitative diagnostic approach for clinical practice. Previous researches have demonstrated the utility of LN-based radiomics in assessing LNM in RC [27]. Song et al. conducted radiomic analysis on small LNs, averaging 4-6 mm in size, and found that model constructed by segmenting regions of interest along the border of LNs exhibited the highest diagnostic efficacy, with an AUC of 0.82 in the test set [28]. In comparison to the complex processes of radiomics, our study established a predictive model based on the Node-RADS score of size-prioritized LNs, achieving comparable diagnostic performance through a more straightforward approach.

In RC, the N stage, therapy regimen and prognosis depends on the number of LNs with metastasis. In this study, we explored the relationship of Node-RADS score to the quantity-dependent pathological characteristics related to number of metastatic LNs, and found that Node-RADS score demonstrated strong correlation to the pN stage, LNM number and LNR, regardless of whether size-prioritized or morphology-prioritized LNs were assessed. According to the NCCN guideline, N1a, N1b, N2a, and N2b represent 1, 2–3, 4–6, and ≥ 7 positive LNs, respectively, which indicates an incremental level in the amount of LNM from N1a to N2b stage. Within each T stage, survival shows an inverse correlation with the N stage (N0, N1a, N1b, N2a, and N2b) in RC [29]. The number of positive LNs has been regarded as a critical predictor for the survival of RC patients [30, 31].However, LNR has recently gained recognition as a prognostic factor in RC. A variety of reports found that LNR has significant effect on the prognosis of RC, including overall survival (OS), disease-free survival (DFS) and recurrence-free survival (RFS), which suggest LNR may contribute to guide clinical decision-making [32–35]. Our positive findings on the correlation between Node-RADS score and quantity-dependent pathological characteristics of metastatic LNs raises the possibility that Node-RADS might serve as a prognostic indicator in RC.

When applicating Node-RADS score to TNM staging, scores 1 and 2 should be reported as negative LNM, while scores 4 and 5 should be classified as positive LNM [16]. The classification of score 3 should depend on primary malignancy, however, the Node-RADS system does not provide guidance on how to classify it for RC [16]. In the present study, a Node-RADS score of > 3 for size-prioritized LN could be considered as the best cut-off, with the highest AUC value of 0.826 among all the individual predictors, which indicated that Node-RADS score 3 is more suitable for being classified as negative LNM in RC. Thus, our research findings may enrich the content of the Node-RADS scoring system.

In order to fully exploit medical information and facilitate clinical applications, we constructed a clinical-imaging integration model (i.e., Clinical-Node-RADS nomogram) which combined the Node-RADS score of size-prioritized LN with clinical characteristics for the preoperative assessment of LNM in RC, and achieved the highest prediction performance (AUC = 0.86). It is three clinical factors, including location, histologic grade and cT stage, were incorporated into the nomogram by AIC in this study. According to AIC, a two-sided* p* < 0.05 is considered statistically significant in univariate logistic regression analysis, but the model with the smallest AIC value will be chosen as the best predictive model in stepwise multivariate logistic regression analysis, rather than using the *p*-value of the variables as the reference [24]. In fact, all the above-mentioned clinical factors have been proven to be associated with LNM in RC [36–39]. cT stage evaluates the tumor based on its local infiltration depth. Tumors with higher cT stages typically exhibit deeper infiltration and greater aggressiveness, which increases the risk of cancer cells spreading to the LNs. Poorly differentiated tumor cells proliferate faster and are more invasive, making it more likely for cancer cells to spread to the LNs. Tumor location is also a crucial factor influencing LNM in RC. High rectal tumors are associated with extensive lymphatic drainage pathways, facilitating the spread of cancer cells to the LNs. Furthermore, high rectal tumors may be closer to major LN groups, thus increasing the risk of metastasis. Notably, the combination of these clinical factors significantly improved the performance of Clinical-Node-RADS nomogram on predicting LNM (0.861 vs. 0.826, *p* < 0.001), compared to the best imaging predictor (i.e., Node-RADS score for size-prioritized LN) in our study, which further confirmed the relationship between the above clinical factors and LNM. Therefore, integrating clinical and imaging information can enhance the performance of CT on detecting LNM in RC.

Our present study had several limitations. Firstly, inherent selection bias might be introduced because of the retrospective nature of this single-centre study. Therefore, future prospective studies with larger sample sizes are warranted to validate our findings and minimize such bias. Additionally, external validation through a multicentre prospective study would strengthen the reliability and applicability of the Node-RADS scoring system in clinical practice. Secondly, this study was on a per-patient basis, whereas individual node-to-node matching between imaging and histopathology was not performed. Therefore, specific characteristics of individual lymph nodes may be overlooked, potentially affecting the accuracy of our Node-RADS assessments. Nevertheless, our study focused on predicting whether patients had LNM or not, and the clinical treatment decision is based on the node status on a per-patient basis regardless of the individual nodes. Lastly, our study did not incorporate quantitative analysis techniques such as texture analysis. Texture analysis has been shown to provide valuable insights into LN characteristics based on CT images. However, smaller LNs will be one of the challenges for this kind of analysis. Future research should aim to explore the potential of incorporating texture analysis to enhance the comprehensiveness of our findings.

## Conclusion

In conclusion, our observations indicated that the Node-RADS scoring system outperforms the short-axis diameter on predicting LNM in RC. Size-prioritized LN demonstrates superior predictive efficacy compared to morphology-prioritized LN. The Clinical-Node-RADS nomogram that combines the Node-RADS score with clinical features exhibits the best diagnostic performance. Moreover, a clear relationship was demonstrated between the Node-RADS score and the quantity-dependent pathological characteristics of LNM.

### Supplementary Information

Supplementary Material 1.

## Data Availability

We prefer not to share our patient raw data. However, the datasets used and/or analysed during the current study are available from the corresponding author on reasonable request.

## Abbreviations

AIC: Akaike information criterion
AUC: Area under the receiver operating characteristic curve
LN: Lymph node
LNM: Lymph node metastasis
Node-RADS: Node Reporting and Data System 1.0
RC: Rectal cancer
