# Supplementary material for: Diagnostic performance of Node Reporting and Data System (Node-RADS) for assessing mesorectal lymph node in rectal cancer by CT
Source: BMC Cancer. 2024 Jun 11;24:716. doi: 10.1186/s12885-024-12487-0 (PMC11165899; doi:10.1186/s12885-024-12487-0)
Supplement: Supplementary file 1 — Supplementary Material 1. [file 12885_2024_12487_MOESM1_ESM.docx]

Supplementary Material


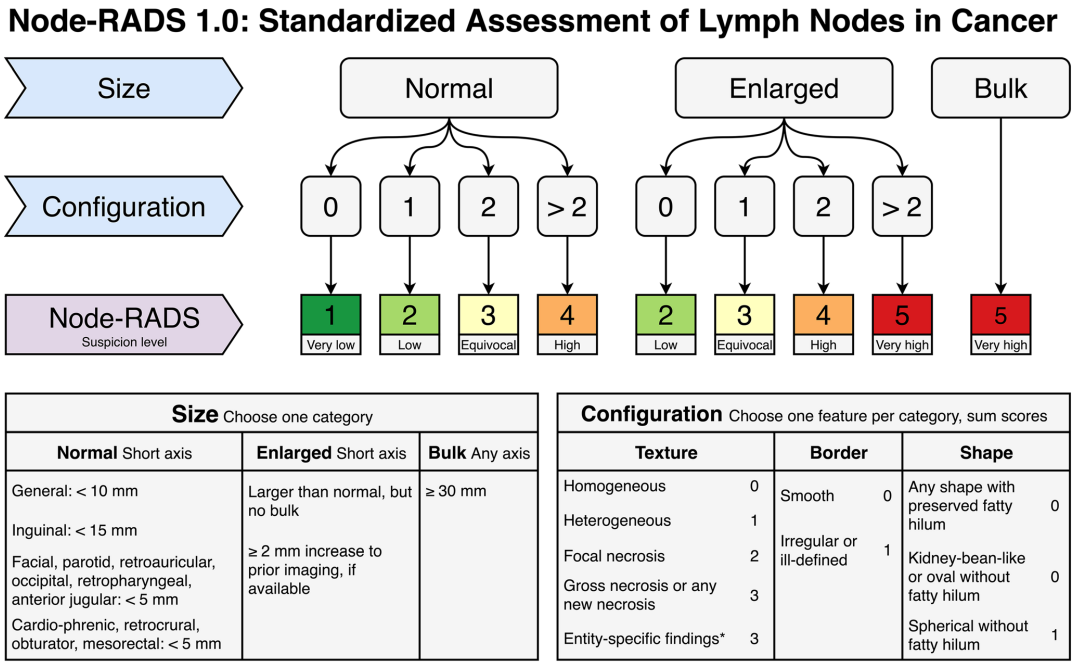


Supplementary Figure S1: Explanation of the Node-RADS scoring system(1). The assessment categories considered level 1 and level 2 addressing size and configuration criteria respectively and level 3 providing Node-RADS score. Size criterion divides LN sizes in normal, enlarged (between normal and bulk criteria) and bulk (longest diameter ≥30 mm). Of note, Node-RADS recommendations provide distinct "normal" size criteria for LN in different locations. Configuration criterion is obtained from the summed numerical values from three sub-categories of texture, border and shape. Texture refers to the internal structure of lymph nodes (homogeneous, heterogeneous, focal necrosis, gross necrosis and entity-specific findings). Border refers to possible extranodal extension of the disease (smooth, irregular or ill-defined). Shape refers to geometric shape and the delineation of fatty hilum (preserved fatty hilum, oval or spheric without fatty hilum). Radiologist must choose one feature from each sub-category with a minimum configuration score of 0 and a maximum of 5. Both "size" and "configuration" contribute to the final LN category. The Node-RADS score range from 1 to 5, reflecting the probability levels of malignancy: "1 - Very low"; "2 - Low"; "3 - Equivocal "; "4 - High"; "5 - Very high."

1. Elsholtz FHJ, Asbach P, Haas M, Becker M, Beets-Tan RGH, Thoeny HC, et al. Introducing the Node Reporting and Data System 1.0 (Node-RADS): a concept for standardized assessment of lymph nodes in cancer. Eur Radiol. 2021;31(8):6116-24.

Supplementary Table S1. Diagnostic performance of different cut-offs of Node-RADS1 score on predicting pN status

| Cutoff | Sensitivity | Specificity | Youden index | PPV | NPV | Accuracy |
| --- | --- | --- | --- | --- | --- | --- |
| >4 | 39.0% | 96.9% | 0.359 | 94.1% | 55.4% | 64.4% |
| >3 | 73.2% | 81.3% | 0.544 | 83.3% | 70.3% | 76.7% |
| >2 | 92.7% | 40.6% | 0.333 | 66.7% | 81.3% | 69.9% |
| >1 | 100.0% | 10.9% | 0.109 | 59.0% | 100.0% | 61.0% |

Abbreviations: Node-RADS1: Node-RADS score of the size-prioritized lymph node; PPV: positive predictive value; NPV: negative predictive value

Supplementary Table S2. Diagnostic performance of different cut-offs of Node-RADS2 score on predicting pN status

| Cutoff | Sensitivity | Specificity | Youden index | PPV | NPV | Accuracy |
| --- | --- | --- | --- | --- | --- | --- |
| >4 | 23.2% | 98.4% | 0.216 | 95.0% | 50.0% | 56.2% |
| >3 | 48.8% | 89.1% | 0.378 | 85.1% | 57.6% | 66.4% |
| >2 | 78.0% | 60.9% | 0.390 | 71.9% | 68.4% | 70.5% |
| >1 | 100.0% | 1.6% | 0.016 | 56.6% | 100.0% | 56.8% |

Abbreviations: Node-RADS2: Node-RADS score of the morphology-prioritized lymph node; PPV: positive predictive value; NPV: negative predictive value

Supplementary Table S3. Diagnostic performance of different cut-offs of Node-RADSmax score on predicting pN status

| Cutoff | Sensitivity | Specificity | Youden index | PPV | NPV | Accuracy |
| --- | --- | --- | --- | --- | --- | --- |
| >4 | 48.8% | 95.3% | 0.441 | 93.0% | 59.2% | 69.2% |
| >3 | 75.6% | 78.1% | 0.537 | 81.6% | 71.4% | 76.7% |
| >2 | 93.9% | 34.4% | 0.283 | 64.7% | 81.5% | 67.8% |
| >1 | 100.0% | 1.6% | 0.016 | 56.6% | 100.0% | 56.8% |

Abbreviations: Node-RADSmax: taking the higher score between Node-RADS1 and Node-RADS2 as the Node-RADSmax score; PPV: positive predictive value; NPV: negative predictive value
